# Enhancing spatial detection accuracy for syndromic surveillance with street level incidence data

**DOI:** 10.1186/1476-072X-9-1

**Published:** 2010-01-18

**Authors:** David J Savory, Kenneth L Cox, Michael Emch, Farrokh Alemi, David C Pattie

**Affiliations:** 1 Planned Systems International, Inc, 5201 Leesburg Pike, Suite 1100, Falls Church, VA, 22041, USA; 2 Armed Forces Health Surveillance Center, 2900 Linden Lane, Suite 200, Silver Spring, MD 20910, USA; 3 Department of Geography and Carolina Population Center, University of North Carolina, Saunders Hall, Campus Box 3220, Chapel Hill, NC, 27599, USA; 4 Department of Health Systems Administration, Georgetown University, 3700 Reservoir Rd, Washington DC 20007, USA

## Abstract

**Background:**

The Department of Defense Military Health System operates a syndromic surveillance system that monitors medical records at more than 450 non-combat Military Treatment Facilities (MTF) worldwide. The Electronic Surveillance System for Early Notification of Community-based Epidemics (ESSENCE) uses both temporal and spatial algorithms to detect disease outbreaks. This study focuses on spatial detection and attempts to improve the effectiveness of the ESSENCE implementation of the spatial scan statistic by increasing the spatial resolution of incidence data from zip codes to street address level.

**Methods:**

Influenza-Like Illness (ILI) was used as a test syndrome to develop methods to improve the spatial accuracy of detected alerts. Simulated incident clusters of various sizes were superimposed on real ILI incidents from the 2008/2009 influenza season. Clusters were detected using the spatial scan statistic and their displacement from simulated loci was measured. Detected cluster size distributions were also evaluated for compliance with simulated cluster sizes.

**Results:**

Relative to the ESSENCE zip code based method, clusters detected using street level incidents were displaced on average 65% less for 2 and 5 mile radius clusters and 31% less for 10 mile radius clusters. Detected cluster size distributions for the street address method were quasi normal and sizes tended to slightly exceed simulated radii. ESSENCE methods yielded fragmented distributions and had high rates of zero radius and oversized clusters.

**Conclusions:**

Spatial detection accuracy improved notably with regard to both location and size when incidents were geocoded to street addresses rather than zip code centroids. Since street address geocoding success rates were only 73.5%, zip codes were still used for more than one quarter of ILI cases. Thus, further advances in spatial detection accuracy are dependant on systematic improvements in the collection of individual address information.

## Background

In the wake of the recent H1N1 pandemic, interest in medical surveillance for early outbreak detection and medical situational awareness continues to grow. Central to this trend are syndromic surveillance systems that employ near real-time monitoring of local or regional clinical records to detect the occurrence of unusual patterns of disease syndromes. Detection methods include both temporal and spatial algorithms with the former typically receiving the most attention. This study focuses on spatial detection and attempts to improve the effectiveness of a commonly used statistic by increasing the spatial resolution of patient location data. The syndromic surveillance system behind this investigation is ESSENCE - Electronic Surveillance System for Early Notification of Community-based Epidemics - which is administered by the Military Health System (MHS) within the Department of Defense (DoD). ESSENCE monitors outpatient visits at non-combat clinics in more than 450 Military Treatment Facilities (MTF) worldwide.

ESSENCE performs daily spatial detection analysis to search for irregular clustering of cases in each of 10 disease syndromes. This study compares the zip code based spatial detection method currently used by ESSENCE with alternative scenarios that vary both the spatial resolution of patient data and statistical nature of the analysis. Using Influenza-Like Illness (ILI) as a test syndrome, the Bernoulli statistical model, street address level patient data, and an alternative background population estimate have been explored as a means of improving the authenticity and spatial accuracy of detected alerts. Accuracy was assessed by superimposing simulated disease clusters on ILI case data and measuring the displacement of detected clusters. Detected cluster size distributions were also evaluated for compliance with simulated cluster sizes.

In a similar study, Olson et al [1] examined the effect of varying levels of address precision on cluster detection by integrating simulated case clusters with actual syndromic surveillance data. Detection accuracy was assessed by considering the proportion of simulated points in identified clusters. In contrast, this study uses the exact location and geometry of identified clusters as a measure of accuracy. Perhaps more significant is the difference in scale - Olson examines a single medical community whereas this study considers regional locales within a global surveillance system. Ozonoff et al [2] investigated spatial detection of simulated data at 12 different levels of aggregation. Again, the proportion of simulated data points in identified disease clusters were used as a measure of accuracy. The proportion of points correctly and incorrectly included in detected clusters was calculated to measure false negative and false positive rates. In both studies, detection accuracy was greatest when exact locations were used and decreased with increasing spatial aggregation. This study attempts to quantify this supposition through spatial analysis. Regarding spatial aggregation, Grubesic and Matisziw [3] provide a detailed analysis of the pitfalls associated with using zip codes for epidemiological analysis.

## Methods

### Spatial Scan Statistic

The spatial detection software used by ESSENCE is adapted from SaTScan, a program developed by Kulldorff [4] which is widely accepted as the de facto standard for spatial-temporal detection of disease clusters. Kulldorff's scan statistics are typically used to detect clusters of disease incidents in both time and space. With ESSENCE, purely spatial methods are used and a non-mathematical description of that statistic is given here. In short, a circular window is scanned across geographic space evaluating the number of observed and expected incidents inside the window at each location. Multiple window sizes are assessed at each location and adjustments are made for the variable density of the background population and the number of cases observed. A cluster is recorded if the null hypothesis is rejected: the spatial distribution of incidents is a random sample from an expected distribution. Ultimately the overall maximum likelihood cluster is determined, i.e., that least likely due to chance. A probability value is assigned to this and any additional clusters detected. Statistics are based on one of several models which include the Poisson and Bernoulli models employed in this study. Details of the statistical theory behind the scan statistic are described further by Kulldorff [5].

ESSENCE spatial detection is based on the Poisson model. Here, the cases at each location are considered to be Poisson distributed, and the expected number of cases is proportional to the population size. This model requires case and population counts for each data location. In ESSENCE, syndrome cases are aggregated by zip code and their centroids are used as the geographic location. Obtaining actual population data typically presents a challenge when relying on medical records since treatment facilities do not serve the entire regional population. This is especially true with MTFs where only military personnel and their families make up the population. One conventional solution known as the Baseline-mean approach [6] utilizes recent historical records to determine expected cases [1]. For example in ESSENCE, data from the 4 week period prior to the analysis date are used to calculate the mean daily syndrome incidents for each zip code [7]. These are adjusted for the day of week and holidays and used as the background population. Note that using street addresses with the Poisson model is problematic given that the statistic requires population data for each case location and households do not have a background population per se.

The Bernoulli model is an alternative scan statistic wherein cases and "non-cases" are analyzed, e.g., patients with ILI symptoms and those without ILI symptoms. These variables are referred to as cases and controls respectively, and their sum is considered the population. Thus, controls can be obtained from records on the date of analysis in contrast to the historical baseline data used in the Poisson implementation. An additional advantage of this model is that it provides for the use of street addresses since cases and non-cases are input at their respective geographic locations.

### Surveillance Data

The clinical dataset is composed of outpatient data from Military Treatment Facilities (MTFs) serving beneficiaries in the state of California. Patient data were obtained from the Clinical Data Mart (CDM) for the 2008/2009 influenza season (September - May). The CDM is a database reporting tool that provides secure access to Armed Forces Health Longitudinal Technological Application (AHLTA), the Department of Defense Military Health System clinical data repository. The clinical focus of this study is the Influenza-Like Illness (ILI) syndrome as defined by ESSENCE standards. An ILI incident is defined as a patient encounter where the patient has been diagnosed with one or more ICD-9-CM code of the set of 12 codes (Table 1) associated with ESSENCE ILI syndrome [7].

**Table 1:** ESSENCE Influenza-Like Illness Syndrome

*ICD - 9*	*Description*
079.99	VIRAL INFECTION NOS

382.9	OTITIS MEDIA NOS

460	ACUTE NASOPHARYNGITIS

461.9	ACUTE SINUSITIS NOS

465.9	ACUTE URI NOS

466.0	ACUTE BRONCHITIS

486	PNEUMONIA, ORGANISM NOS

490	BRONCHITIS NOS

780.6	FEVER

780.60	FEVER, UNSPECIFIED

780.64	CHILLS (WITHOUT FEVER)

786.2	COUGH

Kulldorff's SaTScan software was used to model the customized version of the spatial scan statistic used by ESSENCE. Patient addresses were geo-coded to both the zip code and street level, then analyzed with SaTScan for three detection scenarios including the ESSENCE configuration which serves as the control method (Table 2). Detection of simulated incident clusters with small to medium outbreak extents were performed against the backdrop of real incidents. Cluster radius distributions were also analyzed for comparison with simulated sizes.

**Table 2:** Spatial Detection Scenarios

*SCENARIO*	*Statistical Model*	*Geocode Level*	*Population*
ESSENCE - Zip	Poisson	Zip Code	Baseline-Mean Approach

Bernoulli - Zip	Bernoulli	Zip Code	ILI/Non-ILI

Bernoulli - Street	Bernoulli	Street Address	ILI/Non-ILI

Although patient confidentiality is recognized as an important issue linked to the use of patient addresses, consideration of this issue is beyond the scope of this study. There are numerous studies that address this issue exclusively [8,9]. The authors believe the protection of patient identities to be of utmost importance and that such protection is fully achievable in systems modelled as part of this study.

### Study Area

**Figure 1:**
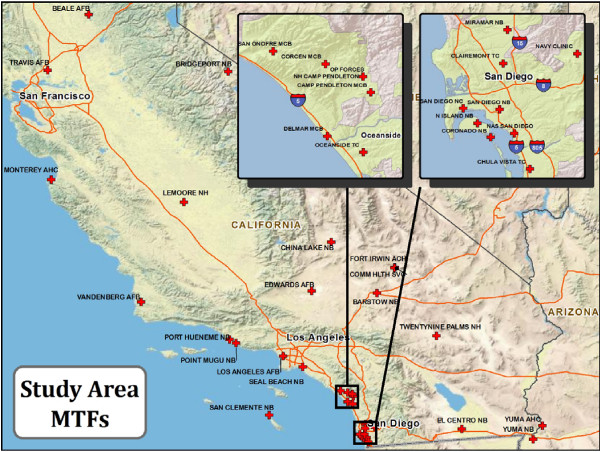
**Study Area**.

The study area for this research was the state of California which is served by 38 MTFs including two clinics in Yuma, Arizona that are in close proximity to the state border (Figure 1). California was selected since it is a large administrative region with MTFs located in a variety of geographies ranging from urban coastal regions to isolated desert and mountain areas. Nearly half of these clinics and hospitals are found in San Diego County in either the southern metropolitan area or in the vicinity of Camp Pendleton to the north. Many of these MTFs have overlapping beneficiary catchment areas, especially in the San Diego metropolitan area. As might be expected, a considerable number of alerts tend to occur in this region. In particular, the clinic at the Naval Branch Health Clinic at the Marine Corps Recruit Depot (MCRD) exhibited an unusually high number of alerts during the research design phase of this study. The combined percentage of ILI incidents at the MCRD and its satellite Edson Annex was 12 times the average of all other MTFs. Given the unique living conditions at recruiting installations that contribute to high rates of incidence, these MTFs have been excluded from the study bringing the MTF total down to 36.

### Resolving Patient Addresses

Ideally, medical surveillance seeks to determine the source of disease outbreaks where it occurs, be it residence or workplace. However due to the nature of available datasets, only home addresses are generally provided including many incomplete or erroneous entries. In this study, most MTF patients are active duty personnel living in close proximity to military installations. However, a considerable number of beneficiaries reside in outlying areas within a couple hours drive. The situation is further complicated by patients away from their primary residence on temporary duty that submit non-local addresses. To resolve this issue, we applied a version of the "100-mile rule" as outlined by Xing et al [6]:

1) Determine the distance of the patient address from the MTF address.

2) The patient zip code or street address was used if the distance was within 100 miles.

3) The MTF zip code or street address was used if the distance was greater than 100 miles.

4) If the home zip code or the street address field was empty or not geocode-able, the MTF zip code/street address was used.

This method was applied to both zip code and street address based scenarios. The main assumption is that an address located more than a short drive from the MTF is a distant permanent address submitted by a patient visiting the local installation. Of the patients in this study, roughly 13% were visiting patients as defined by the 100-mile rule.

### Calculation of Background Populations

The scan statistic requires the calculation of expected incidents at each analysis location and this factor is partially based on the density of the background population. Data streams that reliably provide military and dependent population data are not currently available for use in ESSENCE. The conventional solution used by ESSENCE has been referred to as the Baseline-mean approach [6]. An alternative solution was possible through use of the Bernoulli statistical model and its case/non-case population representation. Methods for making this estimate are detailed by scenario below.

• **ESSENCE - Zip Code**: Population is derived from ILI case data from a 28 day baseline period prior to the analysis date. That is, the mean expected cases for each zip code are calculated from the baseline days of the same day of week as the analysis date. A two day buffer separates the analysis date and baseline period. The purpose of the buffer period is to diminish the effect a current outbreak might have on the baseline statistics [10]. Federal Holidays are grouped with Sundays to model patient behavior. Zip codes with a "population" of zero are set to 1 to comply with scan statistic requirements.

• **Bernoulli - Zip Code**: With the Bernoulli model, the sum of case and control (non-case) counts represents the population. Here, ILI and non-ILI visits are aggregated by zip code for a given analysis date and used as case and control counts, respectively. Non-cases consist of all patient visits that do not contain any of the ICD-9-CM codes mapped to ILI syndrome, i.e., all other visits including, injuries and well-visits.

• **Bernoulli - Street Address**: For this scenario, ILI and non-ILI patient addresses have been geo-coded to the street address level. It is important to note that for many patients, incomplete or erroneous addresses prevent this level of precision in which case home zip codes or the MTF street address is used. Ultimately, the most precise address available was used for all incidents (Table 3). The ILI and non-ILI data points were aggregated by latitude/longitude location for each analysis date and used as case and control counts, respectively.

**Table 3:** Geocoding Success Rates

*Geocode Level*	*Success Rate (%)*	*Success Rate w/100 Mile Rule*
Street Address	80.128	73.491

Zip Code	15.727	13.471

MTF	4.145	4.145

MTF - 100 Mile	---	8.893

### Detection Accuracy Analysis

**Figure 2:**
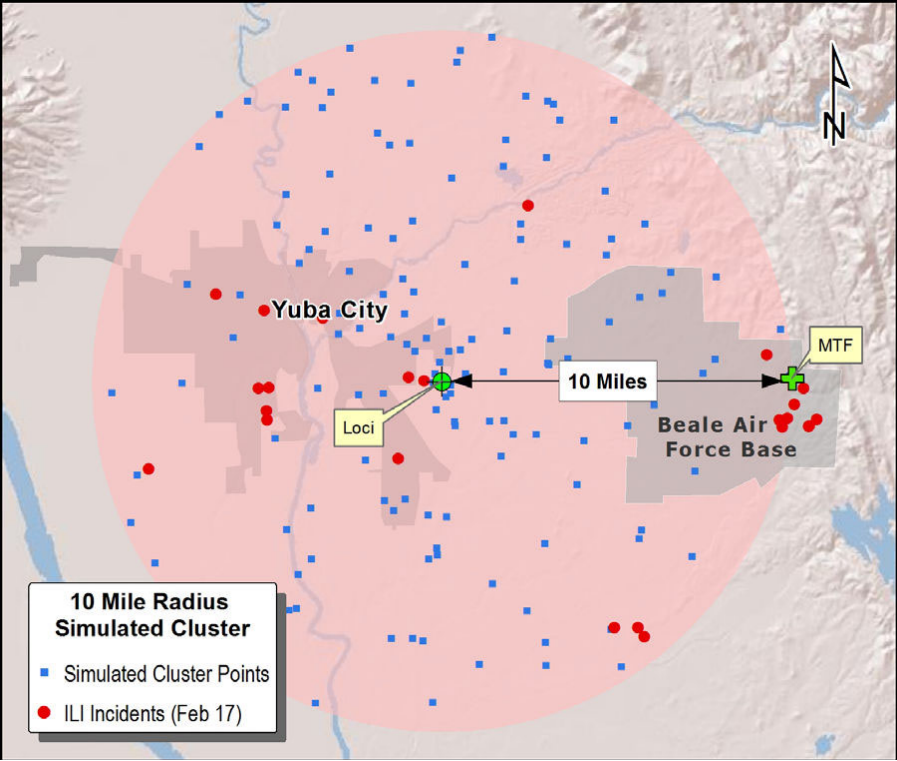
**Cluster Placement Example: 10 Miles West of Beale Air Force Base**.

For purposes of this study, detection accuracy is a measure of how close a detected cluster is to the "true" location of the outbreak. To implement this, detection of simulated incident clusters was performed against the backdrop of real incidents. Since the true center (loci) of simulated clusters is known, the distance to loci from detected clusters can be measured. The CHIP Spatial/Temporal Cluster Generator [11], an open source software application, was used to generate the synthesized clusters consisting of spatially randomized locations. Clusters were positioned within the beneficiary catchment area (40 mile radius) of 10 prominent MTFs representing a variety of geographies within the study area. In large metropolitan areas such as Los Angeles or San Diego, the cluster loci was placed 10 miles due east of the MTF. Otherwise, the loci was placed 10 miles from the MTF in the cardinal direction (i.e., N, NE, etc.) of the most densely populated locality (Figure 2). Such placement adds rigor to the detection testing by ensuring that simulated incidents intermingle with actual ILI cases. Though the number and size of zip codes intersected by simulated clusters impacts the outcome, no attempt was made to influence this factor.

Detection trials included placement of simulated clusters at a 2, 5, and 10 mile radius for each of the 10 loci (Table 4). Although the clusters sizes have different incident densities, they are all sufficiently "conspicuous" for this analysis. SaTScan was run on the amended case data for the 10 MTF locations, 3 cluster sizes, 35 randomly selected dates, and 3 scenarios for a total of 3150 runs. During analysis, ILI cases were defined as those encounters where the diagnosis contained one of the twelve ICD-9 codes that define the ILI syndrome for ESSENCE. Successful detection was defined by clusters that included simulated incidents and conformed to the ESSENCE standard for raising a "red alert". For ESSENCE, red alerts include maximum likelihood or secondary clusters with a scan statistic p-value between 0.001 and 0.01. All instances of this analysis included an ample number of simulated incidents and nearly all were statistically strong red alerts with a p-value of 0.001 (99.87%).

**Table 4:** Simulated Cluster Specifications

*Cluster Radius (mi)*	*Random Incidents*	*Incidents/sq mi*
2	25	2

5	75	1

10	150	0.5

Subsequently, the displacement of detected clusters from the loci was measured. For this measure, detection accuracy is inversely proportional to displacement distance. Detected cluster size distributions were also analyzed for comparison with simulated radii. A close match between the size of original and detected clusters is employed as a second measure of accuracy. As part of the size analysis the rate of zero radius clusters was also recorded. Zero radius clusters, otherwise known as 'singlets', have dubious worth since they represent only a single generalized location.

### Data Processing

The majority of the data processing for this project was accomplished with the relational database and development tools provided by Microsoft Access 2003. Numerous applications were developed using Visual Basic for Applications (VBA) to process the clinical data including resolving patient addresses, extracting ILI cases by location (zip/street address), generating population and control data by location, and formatting and exporting these data for input to SaTScan. Geo-coding to the street address level was accomplished with ArcView Geographic Information System software (ESRI, Inc.). Key SaTScan analysis specifications are listed below.

Type of Analysis: Purely Spatial

Probability Model: Poisson or Bernoulli

Search Locations: Search only from case locations

Maximum Cluster Size: 50% of population at risk/40 miles radius

Criteria for Secondary Clusters: No Geographical Overlap

Post-processing applications were developed for measuring displacement of detected from simulated clusters and production of cluster size distributions. The Haversine formula [12], an equation for measuring spherical distances on the Earth's surface, was used to measure displacement distances.

## Results

To measure accuracy, detection was applied to simulated disease clusters superimposed on ILI case data for 35 randomly selected dates. Detected cluster radius distributions were also analyzed for comparison with actual simulated sizes. In addition, the rate of zero radius clusters was assessed. Comparison of the ability of zip based and street address based methods to correctly detect both the location and size of these clusters provides a relative measure of detection accuracy.

### Cluster Location Analysis

The Bernoulli-Street scenario yielded the most promising results in the location accuracy analysis. Table 5 summarizes the mean displacement of detected clusters for all tested MTFs and dates. Bernoulli-Street clusters were displaced by averages of 1.2, 2.25, and 5.77 miles for 2, 5, and 10 mile radius simulated clusters, respectively. These figures represent improvements in accuracy relative to ESSENCE-Zip of 65% for the 2 and 5 mile radius clusters, and 31% at 10 miles radius. The more modest improvement for the larger clusters is likely due to the increased influence of distant unassociated cases on detection results. Variability was markedly greater in zip code-based methods in general as measured by coefficients of variation. The high standard deviations relative to mean can be explained by the considerable distance between zip code centroids. The results for the Bernoulli-Zip method were similar to that of ESSENCE-Zip.

**Table 5:** Simulated Cluster Displacement Statistics

*Statistic*	*Cluster Radius (mi)*	*ESSENCE-Zip*	*Bern-Zip*	*Bern-Street*
	**2**	3.45	4.24	1.20
	
Mean	**5**	6.49	6.54	2.25
	
	**10**	8.38	8.94	5.77

Coefficient of Variation (*c*_*v *_= *σ/μ*)	**2**	0.63	0.60	0.38
	
	**5**	0.42	0.49	0.54
	
	**10**	0.58	0.44	0.38

**Figure 3:**
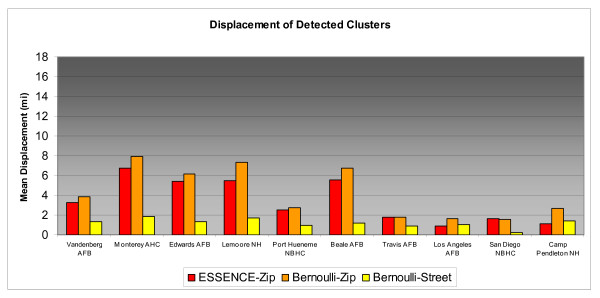
**Displacement of Detected 2 Mile Radius Simulated Clusters by MTF**.

**Figure 4:**
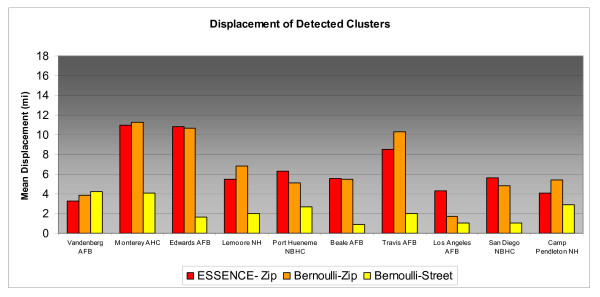
**Displacement of Detected 5 Mile Radius Simulated Clusters by MTF**.

Differences in accuracy results for individual MTFs across the range of simulated cluster sizes can be compared in Figures 3, 4, &5. MTFs in the bar charts are arranged in increasing catchment area beneficiary population from left to right. For 2 and 5 mile clusters, Bernoulli-Street displayed notably better accuracy at nearly all installations. For the larger 10 mile radius clusters street level improvements at many MTFs are less pronounced. The less consistent improvements reflect the influence of distant unassociated cases. Greater displacements may be expected in less populated rural areas due to their typically larger zip code areas. Additionally, military personnel tend to live on the base in rural areas and may be more prone to submitting a non geocode-able installation address during triage. Vandenberg Air Force Base and Lemoore Naval Hospital are two such areas where street level geocoding yielded mixed results, possibly due to these factors. On the right side of the bar chart, the urban based MTFs tended to have more consistent improvements from street level geocoding resolution.

**Figure 5:**
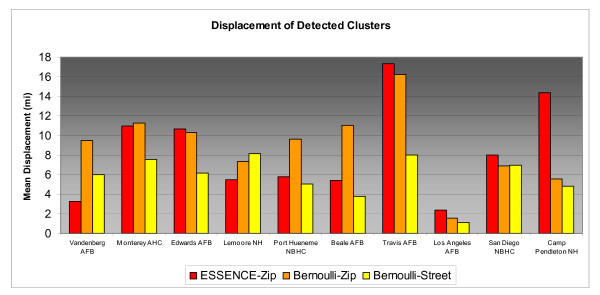
**Displacement of Detected 10 Mile Radius Simulated Clusters by MTF**.

### Cluster Size Analysis

**Figure 6:**
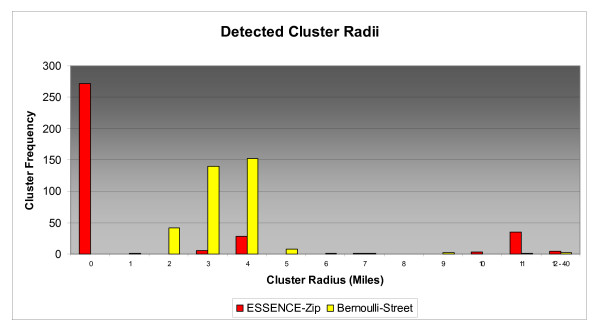
**Size Distribution of Detected 2 Mile Radius Clusters**.

**Figure 7:**
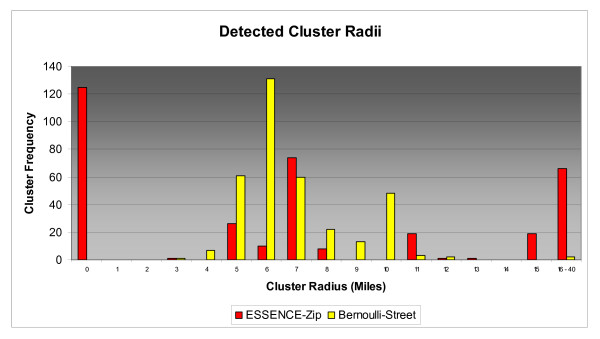
**Size Distribution of Detected 5 Mile Radius Clusters**.

**Figure 8:**
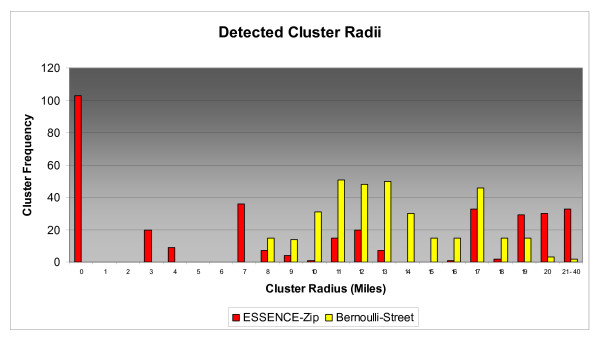
**Size Distribution of Detected 10 Mile Radius Clusters**.

Cluster size distributions varied widely for tested detection scenarios as did the rate of zero radius clusters. Figures 6, 7, &8 depict the size distributions for the ESSENCE-Zip and Bernoulli-Street scenarios and Table 6 gives the rates of zero radius clusters for all scenarios. Although the results for the Bernoulli-Zip scenario were more positive than for ESSENCE-ZIP, they were less favorable than Bernoulli-Street and therefore have been excluded from the histograms in the interest of clarity. In general, distributions were not normally distributed so measures of central tendency have been omitted in lieu of visual analysis of histograms. At 2 miles radius Bernoulli-Street clusters were normally distributed mainly between 2 and 5 miles radius (Figure 6). In contrast, 78% of ESSENCE-ZIP generated clusters had a zero radius. For 5 miles radius, Bernoulli-Street clusters were bi-modally distributed between 3 and 13 mile radius (Figure 7). For ESSENCE-ZIP, although roughly 40% of clusters were in the same range, there were 36% zero radius and 24% with radii of 15 miles or larger. Finally at 10 miles radius, Bernoulli-Street clusters maintained a bimodal but cohesive spread between 8 and 20 miles radius (Figure 8). ESSENCE-ZIP maintained its unacceptable rate of zero radius clusters at 29% with the remainder widely scattered.

**Table 6:** Rate of Zero Radius Clusters at Simulated Radii

*SCENARIO*	*2 Mile Radius*	*5 Mile Radius*	*10 Mile Radius*
ESSENCE - ZIP	0.78	0.36	0.29

Bernoulli - Zip	0.53	0.27	0.03

Bernoulli - Street	0	0	0

## Discussion

The increasing use of geo-spatial technologies in public health and epidemiology has made geocoding - the process of assigning approximated geographic coordinates to address data - a common data processing operation. Consequently, the quality of geocoding methods and its effect on analytical outcomes has become a concern. Issues such as geocoding accuracy, success rates, and address data quality can substantially affect or even drive conclusions drawn from spatial analysis [13,14]. The impetus behind this study was the need to increase the geographic specificity of cluster detection methods commonly used in syndromic surveillance. Increasing the spatial resolution of geocoding methods from the zip code to the street address level was tested towards this end. Interestingly, further improvement of geocoding methods is ultimately what is needed to realize viable street level spatial detection.

### Cluster Location Analysis

The Bernoulli-Street scenario yielded the most promising results in the location analysis with improvements in accuracy of 65% relative to ESSENCE-Zip for the 2 and 5 mile radius clusters, and 31% at 10 miles radius. The larger clusters displayed more modest improvements due to the influence of distant unassociated cases on detection results. It is noteworthy that the results for the Bernoulli-Zip method did not differ significantly from that of ESSENCE-ZIP. This indicates that zip code based spatial detection yields less than optimal spatial accuracy regardless of scan statistic probability model.

Analysis results also indicated that greater displacements, and therefore reduced detection accuracy, may occur in rural areas where zip code areas are larger. An additional confounding issue noted at rural installations is the tendency for military personnel to live in close proximity on base and submit non geo-codable building information as their address. Urban based MTFs responded more consistently to the Street Bernoulli Method since zip codes are smaller and populations more dispersed.

### Cluster Size Analysis

In general, the Street-Bernoulli cluster distributions were quasi normal and sizes slightly exceeded the simulated radii. The offset of the distributions are to be expected since actual incident data surrounding the simulated clusters naturally expands detected sizes. On the contrary, ESSENCE-ZIP distributions displayed fragmented distributions, at least partially due to the incidental spacing of zip code centroids. They also had unacceptably high rates of zero radius clusters, known as 'singlets'. These have dubious worth since they represent only a single generalized location within one zip code area. Surveillance alerts that are raised as a result of singlets or relatively large clusters tend to be taken less seriously than small to moderate size clusters. No singlets were found in analysis results for the Street-Bernoulli method.

### Enhanced Geo-coding

In summary, the measures employed for this study indicate that the Bernoulli-Street scenario displayed the best detection accuracy with regard to both location and size. Displacement of detected clusters from simulated loci was dramatically reduced when the street level incident data was used with the Bernoulli statistical model. Cluster size distributions were also more favorable and than with both zip code based test scenarios.

Improving geo-coding accuracy and success rates may further enhance the accuracy of street level spatial detection. Since ESSENCE-ZIP uses zip code level geocoding, it tends to concentrate incidents at zip code centroids. This incident "stacking" contributes to the high rates of zero radius clusters. A significant amount of stacking occurs even with street addresses, i.e., zip code centroids are used if geo-coding fails or the patient is not a local resident (100 mile rule). Given that street address geocoding success rates were only 73.5%, zip codes were used for more than one quarter of mapped incidents.

## Conclusions

Further improvements in spatial detection accuracy are dependant on systematic improvements in the collection of individual-level address information. Individual data utilized by MTFs are generally captured by the Defense Enrollment Eligibility Reporting System (DEERS) and pushed to AHLTA on a monthly basis. Patients should be encouraged to submit accurate home street addresses in a strict standardized format during enrollment. Visiting MTF patients present a dilemma since their permanent address is automatically used. Ideally, provision should be made for visiting patients to submit a workplace or lodging address in the interest of successful disease surveillance. The best case scenario would be to record both work and residential addresses in all cases, but presently this may be difficult to implement. Lastly, on-base residents must be encouraged to submit geo-codable street addresses rather than building or barrack names. Enhanced address records would improve geocoding success rates resulting in less reliance on zip code centroids. Consequently, the intuitive effect of street address level geo-coding is realized: true incident spatial patterns emerge and the location and size of detected clusters are more accurate.

## Competing interests

The authors declare that they have no competing interests.

## Authors' contributions

DJS conceived of the study and its design, acquired data, developed analytical software, implemented data analysis, and drafted the manuscript. KLC authorized acquisition of data, participated in the study design and coordination, and helped to draft the manuscript. ME participated in the study design and helped draft the manuscript. FA participated in the study design and helped draft the manuscript.

DCP helped draft the manuscript. All authors read and approved the final manuscript.

## Authors' information

During the initial stages of this study Dr. Kenneth L. Cox served as a Colonel in the U.S. Air Force, Director of Global Health Surveillance - Force Health Protection & Readiness (FHP&R), and Chief Functional Proponent for ESSENCE. FHP&R is a program of the Office of the Assistant Secretary of Defense (DASD) - Health Affairs and is the sponsor of this research.
